# Standardizing ototoxicity monitoring for children treated with cisplatin: an urgent call to action

**DOI:** 10.3389/fonc.2026.1923166

**Published:** 2026-10-27

**Authors:** Kristin Knight, Milanka Drenovak, Thorsten Langer, Nienke Streefkerk, Tri Juda Airlangga, Murti Andriastuti, Asmarinah, Deborah Berndtson, Adwina Nurlita Kusuma Wardhani Hartantyo, Alexander E. Hoetink, Kim E. de Jager, Dawn Konrad Martin, Cimi Ilmiawati, M. Tayyar Kalcioglu, Ika Dewi Mayangsari, Nyilo Purnami, Shally Adhina Putri, Haldun Oguz, Irene Schmid, Rasjid Soeparwata, Helen Spoudeas, Anna-Katharina Rohlfs, Kaukab Rajput, Penelope Brock, Marry M. van den Heuvel-Eibrink, Antoinette am Zehnhoff-Dinnesen

**Affiliations:** 1 Pediatric Audiology, Department of Pediatrics, Doernbecher Children’s Hospital, Oregon Health & Science University, Portland, OR, United States; 2 The Royal National ENT and Eastman Dental Hospitals, UCLH NHS Foundation Trust, London, United Kingdom; 3 Pediatric Hematology and Oncology, Department of Pediatric and Adolescent Medicine, University of Lübeck, Lübeck, Germany; 4 Princess Máxima Center for Pediatric Oncology, Utrecht, Netherlands; 5 Department of Otorhinolaryngology, Head & Neck Surgery, Faculty of Medicine, Cipto Mangunkusumo Hospital, University of Indonesia, Jakarta, Indonesia; 6 Pediatric Oncology/Hematology, Department of Pediatrics, Faculty of Medicine, Cipto Mangunkusumo General Hospital, University of Indonesia, Jakarta, Indonesia; 7 Department of Medical Biology, Faculty of Medicine, Universitas Indonesia, Jakarta, Indonesia; 8 Fairfax Audiology Sonus, Fairfax, VA, United States; 9 Pediatric Oncology/Hematology, Faculty of Medicine, Dr. Soetomo General Academic Hospital, Universitas Airlangga, Surabaya, Indonesia; 10 Department of Otorhinolaryngology and Head & Neck Surgery, University Medical Center Utrecht, Utrecht, Netherlands; 11 UMC Utrecht Brain Center, Utrecht, Netherlands; 12 Veterans Affairs Portland Healthcare System, National Center for Rehabilitative Auditory Research, Portland, OR, United States; 13 Department of Otolaryngology, Head and Neck Surgery, Oregon Health & Science University, Portland, OR, United States; 14 Division of Environmental Toxicology, Department of Pharmacology, Faculty of Medicine, Andalas University, Padang, West Sumatra, Indonesia; 15 Department of Otorhinolaryngology & Head and Neck Surgery, Istanbul Medeniyet University, Istanbul, Türkiye; 16 Department of Otolaryngology, Head and Neck Surgery, Faculty of Medicine, Dr. Soetomo General Academic Hospital, Universitas Airlangga, Surabaya, Indonesia; 17 Department of Otolaryngology, Faculty of Medicine, Lokman Hekim University, Ankara, Türkiye; 18 Oncological-Hematological Day Hospital, Dr. von Hauner Children’s Hospital, Munich, Germany; 19 Faculty of Medicine, University of Münster, Münster, Germany; Faculty of Medicine, Universitas Indonesia, Jakarta, Indonesia; 20 Success Life After Cure Ltd, London, United Kingdom; 21 Section of Phoniatrics and Pedaudiology, Department of Otorhinolaryngology, Head and Neck Surgery, University Medical Center, Ulm, Germany; 22 Great Ormond Street Hospital for Children NHS Foundation Trust, London, United Kingdom; 23 Department of Phoniatrics and Pedaudiology, University Hospital, University of Münster, Münster, Germany

**Keywords:** cisplatin, hearing loss, ototoxicity, pediatric oncology, platinum-induced hearing loss

## Abstract

Over the past 25 years, considerable progress has been made in identifying the prevalence, risk factors, functional impacts, and protective strategies for cisplatin-induced ototoxicity. Despite these advances, ototoxicity monitoring and audiologic management of children undergoing cisplatin treatment remains inconsistent across clinical settings. This position paper from the German-Indonesian Society for Medicine (DIGM) Ototoxicity Working Group reflects a consensus process based on expert opinion, supported by a narrative review of the scientific literature and recently published recommendations regarding audiologic management of children receiving cisplatin chemotherapy. We advocate that ototoxicity monitoring be recognized as standard of care for every child receiving cisplatin and incorporated into pediatric cancer treatment protocols. This paper proposes a minimum benchmark for the audiologic management of children undergoing cisplatin chemotherapy; a standard that can be readily implemented in most clinical settings worldwide.

## 1 Introduction

Hearing loss occurs in 50-60% of children that receive cisplatin chemotherapy (1–5), and this condition is permanent. Cisplatin typically impacts the high frequency region of hearing which is essential for the perception of consonant sounds, speech clarity, and understanding speech in noisy environments or at a distance. The risk of hearing loss is greatest, and occurs earlier, in children younger than 5 years at the time of treatment, in those receiving higher individual and cumulative doses of cisplatin, and in those whose treatment also includes cranial irradiation, vincristine, and carboplatin (2–5). Importantly, this risk does not end when treatment is complete. Platinum is known to accumulate and persist within the inner ear (6), which could explain why a progression of hearing loss has been reported in 20-50% of childhood cancer survivors exposed to cisplatin (7, 8). In addition to hearing loss, cisplatin may also cause tinnitus and vestibular dysfunction. However, the prevalence and long-term impact of these ototoxic effects in children have not yet been fully established.

Hearing loss is a lifelong disability that can have considerable implications for an individual’s social participation, economic well-being, and quality of life. Hearing loss that occurs when children are undergoing neurocognitive development has additional developmental and educational impacts including speech, language, and literacy acquisition (9–13). In older children and adolescents, even high-frequency hearing loss can impair listening in everyday environments, negatively affecting educational attainment, social-emotional development, and self-esteem (14–16). The impact of childhood hearing loss extends into adulthood, where it has been associated with reduced employment opportunities, lower income, impaired career progression and diminished lifetime earnings (17, 18).

Pediatric oncologists recognize the risks and impact of cisplatin ototoxicity and consider it an important clinical problem (19, 20). International pediatric clinical guidelines, including those from the International Late Effects Guideline Harmonization Group, the International Society of Pediatric Oncology (SIOP), and the Children’s Oncology Group (COG) recommend that children exposed to cisplatin have a baseline audiologic evaluation before initiation of ototoxic therapy, monitoring during treatment to identify early changes in hearing, and an end-of-treatment evaluation within 3 months after completing platinum chemotherapy (21–23). These guidelines also provide detailed pediatric age-based protocols for audiologic assessments, and specific recommendations for the frequency of monitoring, both during treatment and throughout survivorship (4, 21–23).

## 2 Discrepancies in ototoxicity monitoring guidelines and clinical practice

Despite these advances, ototoxicity monitoring and management remain outside the standard of care for children treated with cisplatin, even in high-resource healthcare settings. This results in underdiagnosed and undertreated hearing loss.

Cohort studies indicate that 19-58% of children treated with ototoxic cancer therapy do not have audiologic monitoring during and at the end of treatment (24, 25). In one series of 185 children treated with platinum chemotherapy and/or cranial radiation, only 43% received hearing assessments before, during, and at completion of therapy (24). In a separate cohort of 305 children exposed to ototoxic therapy, 19% had audiologic monitoring during treatment but not at the completion of therapy, and fewer than half received audiologic follow-up in survivorship (25). Children were less likely to have ototoxicity monitoring if they were not enrolled in a clinical treatment study or when the treatment regimen did not include recommendations for audiologic assessments (25).

The German-Indonesian Society for Medicine (DIGM) Ototoxicity Working Group advocates that ototoxicity monitoring should be recognized as standard of care for every child treated with cisplatin and included within pediatric cancer treatment protocols.

## 3 Recommendations for ototoxicity monitoring for children treated with cisplatin

1. At a minimum, a baseline and an end-of-treatment audiologic assessment should be mandated in every treatment regimen that includes cisplatin.

- *Baseline assessment:* A comprehensive audiologic evaluation should be performed before the initiation of cisplatin, or as early as possible, to establish pre-treatment hearing status (21).
- *End-of-treatment assessment:* a comprehensive audiologic evaluation should be completed within 3 months of the final cisplatin dose, even if the treatment plan is modified or if maintenance therapy continues (21, 22).

Settings with greater resources may be able to provide care that exceeds this minimum standard. Ideally, children treated with cisplatin should have audiologic monitoring during treatment. Enhanced audiologic monitoring is especially important if the child is at increased risk for hearing loss due to age and/or high cumulative cisplatin dose, concurrent therapies, or other risk factors. Preventative strategies to reduce the risk of hearing loss should be utilized whenever possible (26–29).

The protocols in **Table 1**, drawn from the International Society of Pediatric Oncology Supportive Care Consensus Report (21), were developed to provide practical guidance for international multi-center clinical trials; however, more extensive testing is desirable and preferable where feasible.

**Table 1:** Suggested pediatric ototoxicity monitoring protocols for children treated with cisplatin: (4, 21).

Age group	Baseline & end of therapy	Monitoring
≥ 5 years	Audiometry: - Pure tone thresholds^a^ (0.5–8 kHz) - Extended high frequencies (>8 kHz), if available - Speech recognition testing^b^ Otoacoustic emissions: DPOAEs Middle ear: Otoscopy, immittance^c^ Other: Question presence of tinnitus or use tinnitus questionnaire	Audiometry: - Pure tone thresholds^a^ (0.5–8 kHz) - Extended high frequencies (>8 kHz), if available Otoacoustic emissions: DPOAEs Middle ear: Otoscopy, immittance Other: Question presence of tinnitus
7 months -5 years	Audiometry: - Pure tone thresholds^a,d^ (0.5–8 kHz) - Extended high frequencies (>8 kHz), if possible - Speech recognition, if possible Otoacoustic emissions: DPOAEs Middle ear: Otoscopy, immittance^c,e^	Audiometry: Pure tone thresholds^a,d^ (0.5–8 kHz) - Extended high frequencies (>8 kHz), if possible Otoacoustic emissions: DPOAEs Middle ear: Otoscopy, immittance^e^
Infants or children with limited responsiveness	Electrophysiology: - Neurodiagnostic ABR - Tone-burst ABR or ASSR at 1, 2, 4 kHz, and 6 or 8 kHz if possible Otoacoustic emissions: DPOAEs Middle ear: Otoscopy, immittance^c,e^	Otoacoustic emissions: DPOAEs Middle ear: otoscopy, immittance^e^ Electrophysiology: Repeat tone-burst ABR or ASSR if OAE results indicate possible hearing loss or require treatment decisions Audiometry: As soon as able

kHz, kilohertz; DPOAEs, distortion product otoacoustic emissions; ABR, Auditory brainstem response; ASSR, Auditory steady state response.

^a^ Include bone conduction measurement when thresholds are >20 dB HL, and/or when immittance measures indicate middle ear dysfunction.

^b^ Speech recognition testing in quiet and noise when possible.

^c^ Include acoustic reflex measurement or screening for children who have CNS cancers.

^d^ Audiometry method based on the child’s age and development (standard, conditioned play, visual reinforcement).

^e^ Use 1 kHz probe tone for immittance testing for infants younger than 9 months old.

Baseline: Before cisplatin therapy starts or as soon as possible after the first cycle.

Monitoring: audiologic testing during treatment, frequency based on cisplatin dose per cycle:.

>50 mg/m2: every cisplatin cycle.

<50 mg/m2: every other cisplatin cycle if no other risk factors (no planned exposure to carboplatin, vincristine, cranial radiation).

Child with baseline hearing or vision loss: every cycle, any exposure.

End of Therapy: 4–12 weeks after completion of ototoxic therapy.

2. Hearing evaluations or hearing screening assessments should be integrated into routine follow-up care.

Late-onset and progressive hearing loss can impact the patient long after treatment. Audiology should be included in childhood cancer survivorship programs so that patients can access long-term hearing management and resources. The International Late Effects of Childhood Cancer Guideline Harmonization Group (22) and the Children’s Oncology Group (23) have developed recommendations for audiologic management after treatment, and these are summarized in **Table 2**.

**Table 2:** Long-term follow-up guidelines after cancer treatment, pediatric through young adult: (22, 23).

Exposure	Dose	Timing of hearing evaluation
Cisplatin	Any	Child age: ≤ 6 years: yearly 6–12 years: every two years ≥ 12 years: every five years • If hearing loss identified, repeat yearly. • If there is evidence of progressive hearing loss, perform more frequent audiologic assessments, as recommended by hearing specialist, until hearing thresholds stabilize.
Carboplatin	Myeloablative doses (>1500 mg/m2), or any dose if age at exposure was < 1 year	
Cranial/brain radiation therapy	≥30 Gy	

Considerations:.

Audiology consultation for any survivor who has symptoms of hearing loss, tinnitus, or an abnormal hearing screen.

Ongoing follow-up with audiology when hearing loss (>20 dB HL 0.5–8 kHz) is identified.

Otolaryngology consultation for patients with chronic ear infection, cerumen impaction, or anatomical conditions contributing to hearing loss.

## 4 Discussion

International guidelines converge on this core monitoring framework: baseline audiological evaluation before ototoxic therapy, monitoring during treatment, and an end-of-treatment assessment within 3 months of the final cisplatin dose (21–23, 30). Surveys and cohort studies have identified barriers that impede the implementation of ototoxicity monitoring. These include a lack of structural integration within healthcare systems, economic constraints, limited healthcare resources, and logistical challenges (30–40). Other related obstacles to implementation include the absence of audiology services within or near cancer treatment centers, inflexible scheduling, and reliance on families to manage referrals and coordinate care (31–33, 40). Ototoxicity monitoring should therefore be embedded as a predefined component of the cancer treatment pathway rather than functioning as a referral-dependent service requiring initiation and coordination by individual clinicians or families.

Key facilitators that can help overcome barriers to ototoxicity monitoring include having well-defined protocols, strong systems for interdepartmental communication, use of multidisciplinary teams to co-develop processes, access to portable diagnostic equipment, systems for ensuring completion of key clinical endpoints such as baseline and post-treatment assessments, and a dedicated ototoxicity management program (30, 34–40). At the operational level, clearly assigned responsibilities, predefined assessment time points, standardized electronic prompts or order sets, and systems for tracking pathway adherence and missed assessments may further reduce variability and loss to follow-up. Institutional commitment, including policy development and adequate reimbursement, is also necessary for sustainable program delivery.

It can be challenging to obtain ear-specific behavioral hearing results in infants and young children. Prioritizing high frequency measurement, using a team-testing approach, and including objective measures have been recommended (21, 41, 42). The “cross-check” principle combines behavioral audiometric tests, which require the child’s active participation, with objective measures that assess auditory function (43). When behavioral testing isn’t feasible, objective measures are essential. Hearing thresholds can be estimated with auditory brainstem response (ABR) and/or auditory steady state response (ASSR), ideally including frequencies above 4 kHz. These can be conducted during natural sleep, or with sedation or anesthesia.

The International Ototoxicity Management Group (IOMG) has developed a comprehensive framework with strategic priorities to support global implementation of ototoxicity management services for patients undergoing ototoxic cancer therapy across the lifespan (30). The DIGM highlights this work and endorses the recommendations. Establishing a minimum benchmark standard for ototoxicity monitoring within pediatric cancer protocols by pediatric oncology groups would provide essential structural support and accountability for the implementation of ototoxicity management programs. Oversight by oncology governing bodies would promote institutional commitment and resource allocation, facilitate broader adoption of these programs, and ultimately improve hearing outcomes in this vulnerable population. Only once this basic standard is met can healthcare institutions be compelled to provide care needed to effectively apply crucial preventative strategies to reduce the risk of hearing loss or address its adverse impacts (26–29).

## Data Availability

The original contributions presented in the study are included in the article/supplementary material. Further inquiries can be directed to the corresponding authors.
